# Hæmorrhage from the Uterus after the Menopause

**Published:** 1907-05-25

**Authors:** H. T. Hicks

**Affiliations:** Assistant Surgeon to the Samaritan Hospital, Obstetric Registrar to Guy's Hospital


### GYNÆCOLOGY.

### HÆMORRHAGE FROM THE UTERUS AFTER THE MENOPAUSE.

By H. T. HICKS, Assistant Surgeon to the Samaritan Hospital, Obstetric Registrar to Guy's Hospital.

IRREGULAR hæmorrhage at or about the time of the menopause is a matter of almost everyday experience, and may be due to innocent or malignant new growth. More often, however, it is associated with the vascular changes which take place in the uterus at this time. Arterial degeneration plays an important part in the causation of irregular uterine hæmorrhage, so we find that patients suffering from chronic nephritis or chronic alcoholism sometimes bleed profusely, especially when the vascular changes are taking place at the menopause.

When, however, the menopause is once definitely passed the uterus atrophies and ceases to be a functional organ. Hæmorrhage then is seldom due to simple vascular causes, but rather to new growth. We will not discuss growths of the cervix, which are very obvious causes of hæmorrhage, but shall consider the difficulties which are met with in dealing with hæmorrhage from the body of the uterus.

Fibroids, especially submucous tumours, will cause bleeding after the menopause, but as a rule there is a history of previous menorrhagia, and the bleeding with fibroids does not often recur when the menopause is definitely passed. Large fibromyomata may remain quiet for a considerable period after the menopause and then degenerate or become sarcomatous and give rise to hæmorrhage. This is well illustrated in the following case:—

A lady, aged 55, was known to have had a fibroid about the size of a cocoa-nut for nine years. The menopause was reached at the age of 52 years. Two-and-a-half years later, and six months before the operation, she began to bleed from the uterus, the tumour became soft and increased in size. At the operation a large red necrotic tumour (necrobiosis) was removed.

Fibromyomata must be looked upon as a rare cause of bleeding after menstrual life has definitely ceased, and if hæmorrhage does occur in connection with a fibromyoma, carcinoma of the endometrium should be suspected. It is by no means uncommon for a carcinoma to develop in a fibroid uterus, and as an example of this I may briefly refer to a case from the out-patient department of the Samaritan Hospital. A woman, aged 52, had ceased menstruating for two years. Previous to the menopause there had been fairly profuse menorrhagia. On examination a fibromyoma of about the size of an orange was found, growing in the uterine fundus. One might have been tempted to diagnose that the bleeding was due to the fibroid. I curetted the uterus, and found that the curettings were obviously carcinomatous. Subsequently I removed the uterus, and the patient was well a year after the operation. There is no direct relationship between fibromyoma and carcinoma, but these two forms of growth are most often met with in sterile women. Hence their not infrequent association.

Most text-books state that so-called senile endometritis is a cause of a blood-stained watery discharge, but so far as I know, there is no satisfactory pathological evidence which justifies the term, senile endometritis. If a patient is suffering from a watery, blood-stained uterine discharge, she should be looked upon as a case of carcinoma, until exploration fails to demonstrate the disease. It must be remembered that the uterus may be hardly enlarged and yet contain a carcinoma. Tuberculous endometritis may occur late in life, but it is rare and is almost invariably diagnosed as cancer of the body until the uterine curettings are examined.

There are a few other causes of uterine hæmorrhage in late life, such as malignant disease of the tubes and ovaries, but in these there is no great difficulty in diagnosis, owing to the presence of a definite tumour. It is cases of bleeding without apparent cause which give rise to difficulty. The bleeding in carcinoma of the uterus may be watery, or there may be recurrent attacks of more or less profuse hæmorrhage. Sometimes one may find a history of three years' bleeding in this disease, and even after this long period the uterus may not be greatly enlarged and the growth may still be within the limits of the uterine muscle. It is surprising how chronic some forms of carcinoma are and how good the prognosis is after operative interference. In fact, carcinoma of the body of the uterus is one of the least malignant forms of cancer met with in the human body. Metastatic growths are rare, and local dissemination and infection do not occur as a rule till late in the disease. If one contrasts the prognosis of carcinoma of the endometrium with that of carcinoma of the cervix, how hopeful is the former and how hopeless the latter. It seldom happens that carcinoma of the body does not give a timely warning of its existence, and even slight hæmorrhage from the uterus, occurring in later life should always excite suspicion. An exploratory curetting should be advised in all cases of irregular hæmorrhage, and when once carcinoma is diagnosed abdominal pan-hysterectomy should be performed. If the operation is decided upon, great care must be taken to guard against sepsis, for this is perhaps the greatest danger. The uterine cavity is often intensely septic, and so is the vagina, and the peritoneum is very likely to become infected. The immediate dangers following the operation are more serious than are the remote dangers of recurrence. For three days before the operation the vagina should be douched and compressed by means of antiseptic gauze plugs, and immediately before the operation, when the uterus is being removed by the abdominal route, the cervix should be tightly closed with sutures to prevent the escape of septic material.

